# Exploration of biomarkers for systemic lupus erythematosus by machine-learning analysis

**DOI:** 10.1186/s12865-023-00581-0

**Published:** 2023-11-10

**Authors:** Xingyun Zhao, Lishuang Duan, Dawei Cui, Jue Xie

**Affiliations:** 1 Department of Blood Transfusion, The First Affiliated Hospital, Zhejiang University School of Medicine, Hangzhou, China; 2 Department of Anesthesia, The First Affiliated Hospital, Zhejiang University School of Medicine, Hangzhou, China

**Keywords:** Systemic lupus erythematosus, Machine learning, Biomarker, Genes, Databases

## Abstract

**Background:**

In recent years, research on the pathogenesis of systemic lupus erythematosus (SLE) has made great progress. However, the prognosis of the disease remains poor, and high sensitivity and accurate biomarkers are particularly important for the early diagnosis of SLE.

**Methods:**

SLE patient information was acquired from three Gene Expression Omnibus (GEO) databases and used for differential gene expression analysis, such as weighted gene coexpression network (WGCNA) and functional enrichment analysis. Subsequently, three algorithms, random forest (RF), support vector machine-recursive feature elimination (SVM-REF) and least absolute shrinkage and selection operation (LASSO), were used to analyze the above key genes. Furthermore, the expression levels of the final core genes in peripheral blood from SLE patients were confirmed by real-time quantitative polymerase chain reaction (RT-qPCR) assay.

**Results:**

Five key genes (ABCB1, CD247, DSC1, KIR2DL3 and MX2) were found in this study. Moreover, these key genes had good reliability and validity, which were further confirmed by clinical samples from SLE patients. The receiver operating characteristic curves (ROC) of the five genes also revealed that they had critical roles in the pathogenesis of SLE.

**Conclusion:**

In summary, five key genes were obtained and validated through machine-learning analysis, offering a new perspective for the molecular mechanism and potential therapeutic targets for SLE.

**Supplementary Information:**

The online version contains supplementary material available at 10.1186/s12865-023-00581-0.

## Background

Systemic lupus erythematosus (SLE) is an immune-mediated autoimmune disease with clinical manifestations of multisystem damage, often causing irreversible damage to multiple organ systems, seriously affecting the life span of patients [1]. The pathogenesis is complex and it occurs mostly in young women. The abnormal immune system mediated by B cells and T cells is a key link in the occurrence and development of SLE [2–4]. The clinical manifestations of SLE are very diverse, and different individuals exhibit different clinical characteristics at different times. This phenotypic heterogeneity makes the clinical diagnosis and monitoring of disease activity of SLE extremely challenging [5]. Currently, the immunological diagnostic criteria of SLE mainly include anti-dsDNA antibodies, anti-nuclear antibodies and anti-SM antibodies [6]. However, their specificity and sensitivity are low, and the diagnosis efficiency of SLE is still not satisfactory. Hence, exploring the feature genes related to the pathogenesis and development of SLE is necessary.

In recent years, new technologies including next-generation sequencing and mass spectrometry have made great advances in discovering novel biomarkers for the assessment of disease activity and diagnosing of SLE [7, 8]. With the improvements in bioinformatics, various methods are also emerged for the prediction of SLE. However, several bioinformatic methods with accuracy and low efficiency may not enough for screening and early detection of SLE. For example, the traditional differentially expressed genes (DEGs) analysis may result in the loss of intrinsic biological information. At present, machine learning algorithms, combined with other bioinformatics methods, are widely used to screen biomarkers with more diagnostic value.

In this study, we processed DEGs and WGCNA analysis to identify candidate genes associated with SLE. Then, a variety of machine learning algorithms including LASSO, RF and SVM-REF, were combined to obtain the five optimal key genes. Furthermore, ROC was used to evaluate the predictive performance of these genes. Subsequently, GO, KEGG, DO, and GSEA analyses were used to investigate the mechanism of their contribution to SLE. In addition, ssGSEA, an immune-related algorithm, was applied to assess the infiltration levels of different immune cell types. To verify the bioinformatic results, RT-qPCR was used to analyze the relative expression of the five optimal key genes in SLE and healthy control samples. Overall, we found five genes with potentially strong diagnostic effects in SLE patients, suggesting that they might be the new targets for studying SLE.

## Materials and methods

### Download and processing of data

**Fig. 1:**
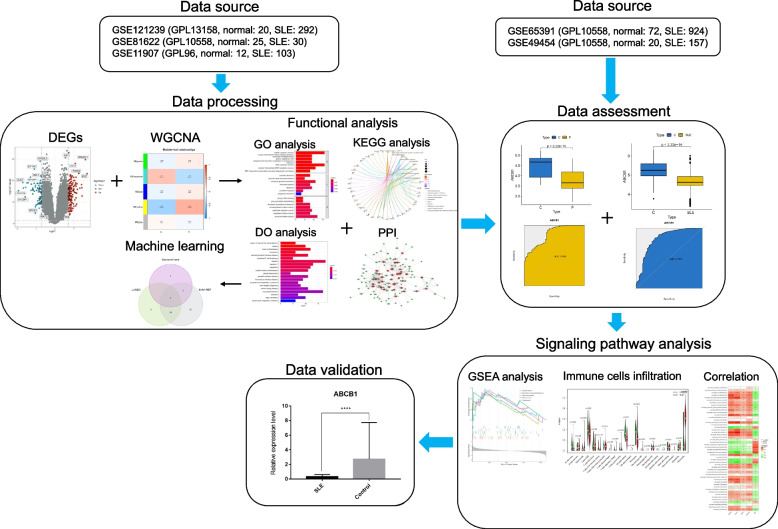
The flowchart of this study

The flowchart is shown in Fig. 1. The raw gene expression data were obtained from the GEO database (https://www.ncbi.nlm.nih.gov/geo/) [9]. Three datasets, GSE121239 (GPL13158, normal: 20, SLE: 292), GSE81622 (GPL10558, normal: 25, SLE: 30) and GSE11907 (GPL96, normal: 12, SLE: 103), were incorporated in the subsequent bioinformatic analysis. Two datasets, GSE65391 (GPL10558, normal: 72, SLE: 924) and GSE49454 (GPL10558, normal: 20, SLE: 157), were used as independent validation sets. The information on these datasets is presented in Supplementary Table 1. The microarray data were converted into log2 values, and the combat algorithm implemented in the R package “sva (version 3.40.0)” [10] was used for integration to remove the batch effect and form a combined dataset [11].

### Differential gene expression analysis

DEGs were identified by differential expression analysis between SLE and control samples through the R package “limma (version 3.50.3)” [12] (criteria: | logFC| > 0.75, adj.*p*.value < 0.05). Volcano plot and heatmap showing the significantly upregulated and downregulated genes.

### Weighted gene coexpression network analysis

The potential functional modules of SLE samples were identified through the R package “WGCNA (version 1.72-1)” [11, 13]. According to the weighted correlation adjacency matrices and cluster analyses, genes with similar expression patterns were allocated to coexpression modules. The topological overlap matrix (TOM) was derived from the adjacency matrix, and genes were assigned into different modules on the basis of the difference between them in the TOM. Notably, the cut height was set to 0.25, the minimal module size was set to 50, and the soft-thresholding power was set as 25 (scale-free *R*^2^ = 0.9). Furthermore, both gene importance (GS) and module membership (MM) were analyzed. The Spearman correlation coefficients and the corresponding *P* values were also analyzed. Finally, the corresponding genes extracted from the hub module were obtained for further analysis.

### GSEA and correlation analysis

GSEA was used to identify the biological significance of optimal key genes [14, 15]. To obtain a normalized enrichment score, each analysis required the gene set permutations with 1000 times. And FDR < 0.05 was considered as a significant enrichment. Additionally, Pearson correlation analysis was utilized to analyze the correlations between optimal key gene expression levels.

### Functional enrichment analysis

Through the above analysis, the overlapping genes between DEGs and module genes were further obtained. A Venn diagram was constructed to show the overlapping genes. The “clusterProfiler (version 4.0.5)” [16] and “DOSE (version 3.20.1)” [17] R packages were used to perform Gene Ontology (GO), Kyoto Encyclopedia of Genes and Genomes (KEGG), and Disease Ontology (DO) enrichment analyses to explore the function and pathways of these genes [18]. Additionally, a protein-protein interaction (PPI) network was created to study the interaction of overlapping candidate genes through “STRING” (https://string-db.org/). The coexpression network was also obtained with the R package “igraph (version 1.4.2)” [19] to study the correlation among these candidate genes. Additionally, the appropriate copyright permission of these KEGG image was obtained and used in this study.

### Further screening of optimal key genes

A total of three machine learning algorithms including LASSO, SVM-REF and RF, were applied to explore the disease status [20–22]. The LASSO algorithm was used to select variables and adjust complexity [23]. The SVM-RFE algorithm was used to analyze the most appropriate key genes [24, 25]. For the RF algorithm, the principle is to find the most reliable results from a large number of underlying tree models [26, 27]. Since the importance of genes greater than 2 in the RF algorithm is a commonly used screening criterion, the top 10 key genes were selected as new gene markers to predict the prognosis of SLE. Finally, the common genes obtained from the intersection of these several algorithms were the optimal key genes.

### The expression and predictive value of optimal key genes

The Wilcoxon rank-sum test was used to analyze the expression levels of optimal key genes. The ROC curve was used to verify the predictive value of the optimal key genes [28, 29].

### Analysis of hallmark gene sets and immune cell infiltration

The CIBERSORT algorithm is a tool that uses a deconvolution algorithm to predict cell proportions, which helps to calculate the cell composition of complex immune tissues based on standardized gene expression data and quantify the abundance of specific cell types [30]. In this study, the CIBERSORT algorithm was used to analyze the infiltration of 22 immune cells in SLE and normal samples. The difference in immune cell proportions was analyzed by the Wilcoxon rank-sum test, and *P* < 0.05 was considered statistically significant. In addition, the ssGSEA algorithm was used to quantify the relative levels of 50 marker gene sets [31]. In addition, Spearman’s correlation between 50 hallmark gene sets and the optimal key genes was also obtained.

### Isolation of human PBMCs

A total of 13 samples were collected from the First Affiliated Hospital, Zhejiang University School of Medicine, including 6 SLE samples and 7 healthy controls. Human peripheral blood mononuclear cells (PBMCs) were isolated by FicollPaque density gradient centrifugation with EDTA anticoagulant blood.

### Real-time quantitative PCR

Total RNA from the PBMCs of SLE patients and healthy controls was extracted with TRIzol reagent, and cDNA was synthesized by reverse transcription with the PrimeScript™ RT Reagent Kit (TaKaRa, China). β-Actin was used as an internal reference. RT-qPCR was performed with the SYBR Green PCR Kit (TaKaRa, China) based on the manufacturer’s protocol [32, 33]. Furthermore, the relative expression was calculated by the 2^−△△CT^ method. All reactions were repeated in triplicate. The primers are displayed in Supplementary Table 2.

### Statistical analysis

R software (version 4.1.1) and GraphPad Prism (version 7.0) were used for data processing, statistical analysis and mapping. The Wilcoxon rank-sum test or Student’s t test identified the differences between two groups. The correlations were analyzed with Pearson’s or Spearman’s correlation tests. *P* < 0.05 was considered statistically significant.

## Results

### DEGs between SLE and control samples

**Fig. 2:**
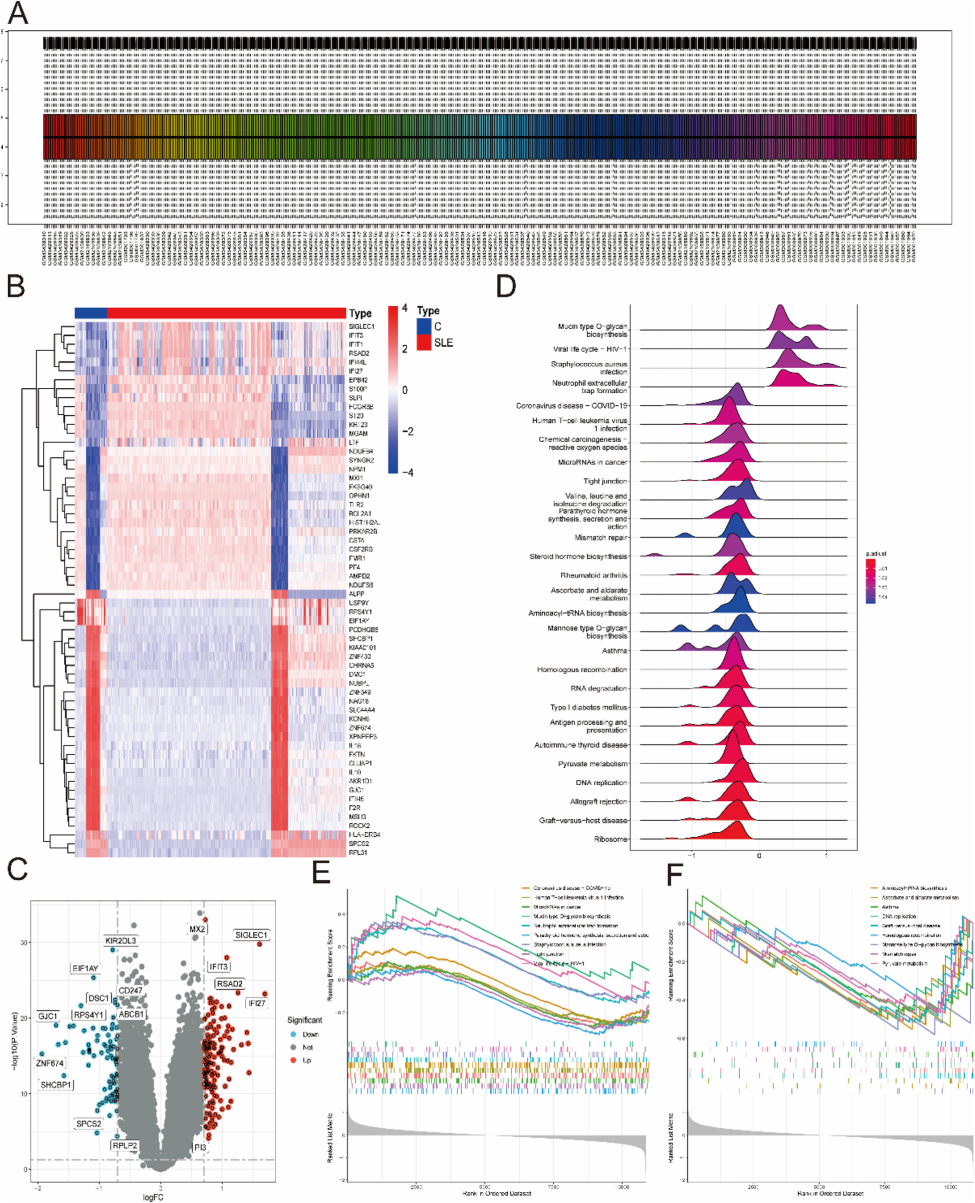
Identification of DEGs between SLE and control samples. **A** The expression level of genes from these datasets after the batch effect removed. **B** The heatmap of SLE-related DEGs expression: blue means low gene expression; red means high gene expression. **C** The volcano plot of SLE-related DEGs expression. **D** The ridgeline map of gene metabolism in SLE. **E**, **F** The main signaling pathways that are significantly enriched in the SLE group (**E**), and in the control group (**F**)

From the GEO database, three microarray datasets, including the GSE121239, GSE81622 and GSE11907 datasets, were combined to obtain a total of 57 control and 425 SLE samples. After the batch effect was removed (Fig. 2A), 242 DEGs (Supplementary Table 3) were identified, including 87 downregulated genes and 155 upregulated genes (Fig. 2B). As shown in Fig. 2C, some genes, such as KIR2DL3, EIF1AY, CD247, ABCB1, DSC1, RPS4Y1, GJC1, ZNF674, SHCBP1, SPCS2, and RPLP2, were significantly upregulated, while some genes were significantly downregulated, such as MX2, SIGLEC1, IFIT3, RSAD2, IFI27, and PI3. Next, GSEA of KEGG was performed to select the enriched signaling pathways (Supplementary Table 4). The changes in metabolism-related genes in SLE, such as pyruvate metabolism, DNA replication and RNA degradation were displayed (Fig. 2D). Notably, mucin type O-glycan biosynthesis, microRNAs in cancer, and tight junctions were significantly enriched in the SLE group (Fig. 2E). However, DNA replication, aminoacyl-tRNA biosynthesis and mismatch repair were significantly enriched in the control group (Fig. 2F).

### Application of WGCNA

**Fig. 3:**
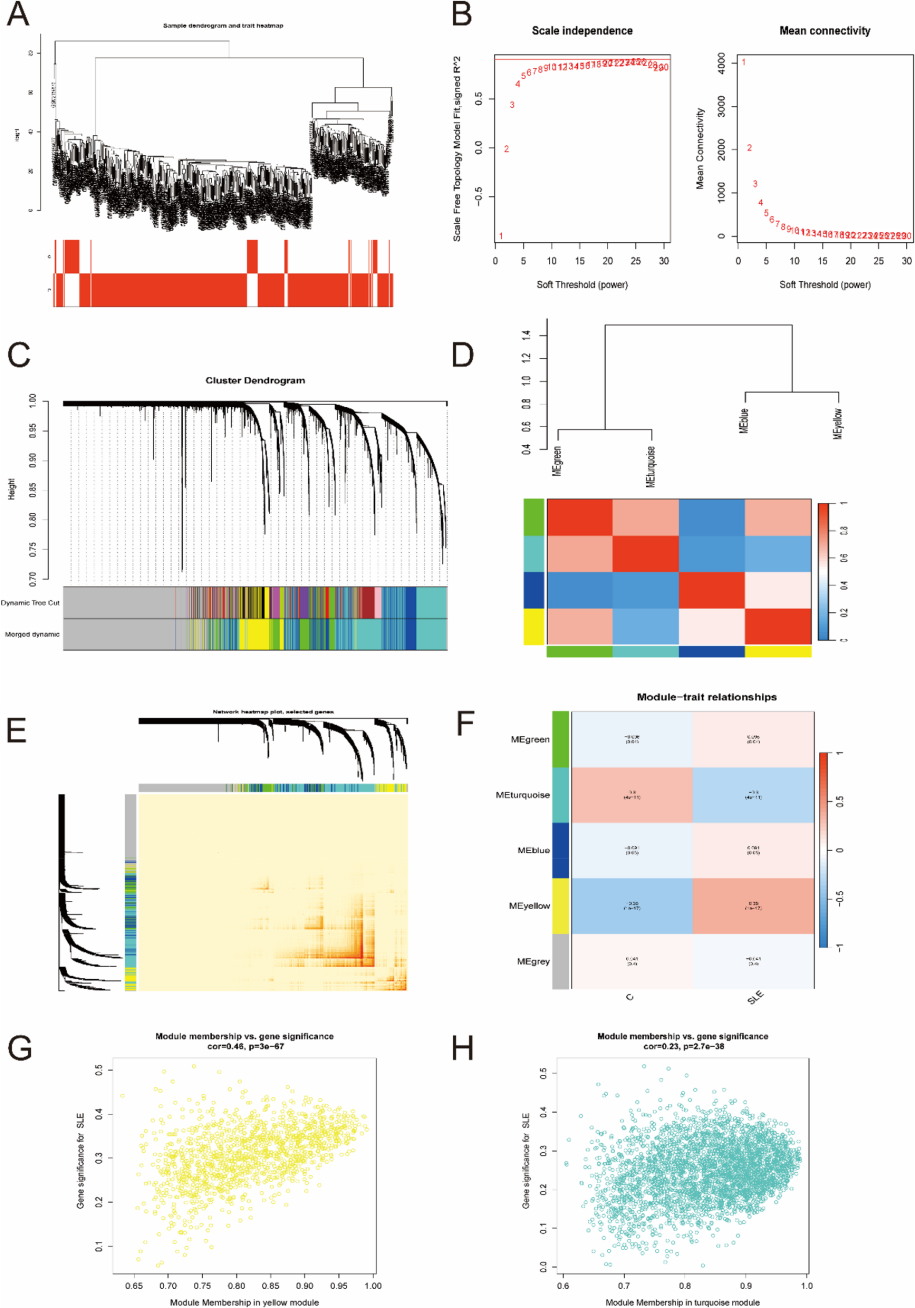
Weighted gene co-expression network analysis. **A** Sample clustering dendrogram with tree leaves corresponding to individual samples. **B** The scale-free fitting index (R2) and average connectivity at different soft threshold power were analyzed. **C** The initialized and integrated modules under the clustering tree. **D** Collinear heat map of module key genes. Red means a high correlation, blue means a low correlation. **E** Clustering dendrogram of module key genes. **F** Heat map of module–trait correlations. Red indicates a positive correlation and blue indicates a negative correlation. **G**, **H** The frontal correlation between clinical features and yellow module (**G**), and turquoise module (**H**)

The coexpression network was established by WGCNA. A total of 11,256 genes were selected for clustering, and obviously abnormal samples were excluded by setting thresholds (Fig. 3A). Then, when *R*^2^ = 0.9 and the average connectivity was high, the soft power threshold was set to 25 (Fig. 3B). After the associated modules were integrated, five modules were obtained. The initialized and integrated modules were finally shown under the clustering tree (Fig. 3C). No significant correlation between the modules was observed (Fig. 3D). In addition, the transcriptional correlation analysis revealed that there was also no relationship between them (Fig. 3E). The frontal correlation was applied to examine the correlation between clinical features and ME values, and the results showed that yellow and blue modules were significantly correlated with SLE (*R* = 0.46, *P* < 3e-67; *R* = 0.23, *P* < 2.7e-38) (Fig. 3F-H). In the yellow and turquoise modules, a total of 4348 candidate genes were obtained in the subsequent analysis (Supplementary Table 5).

### Functional enrichment analysis of overlapping genes

**Fig. 4:**
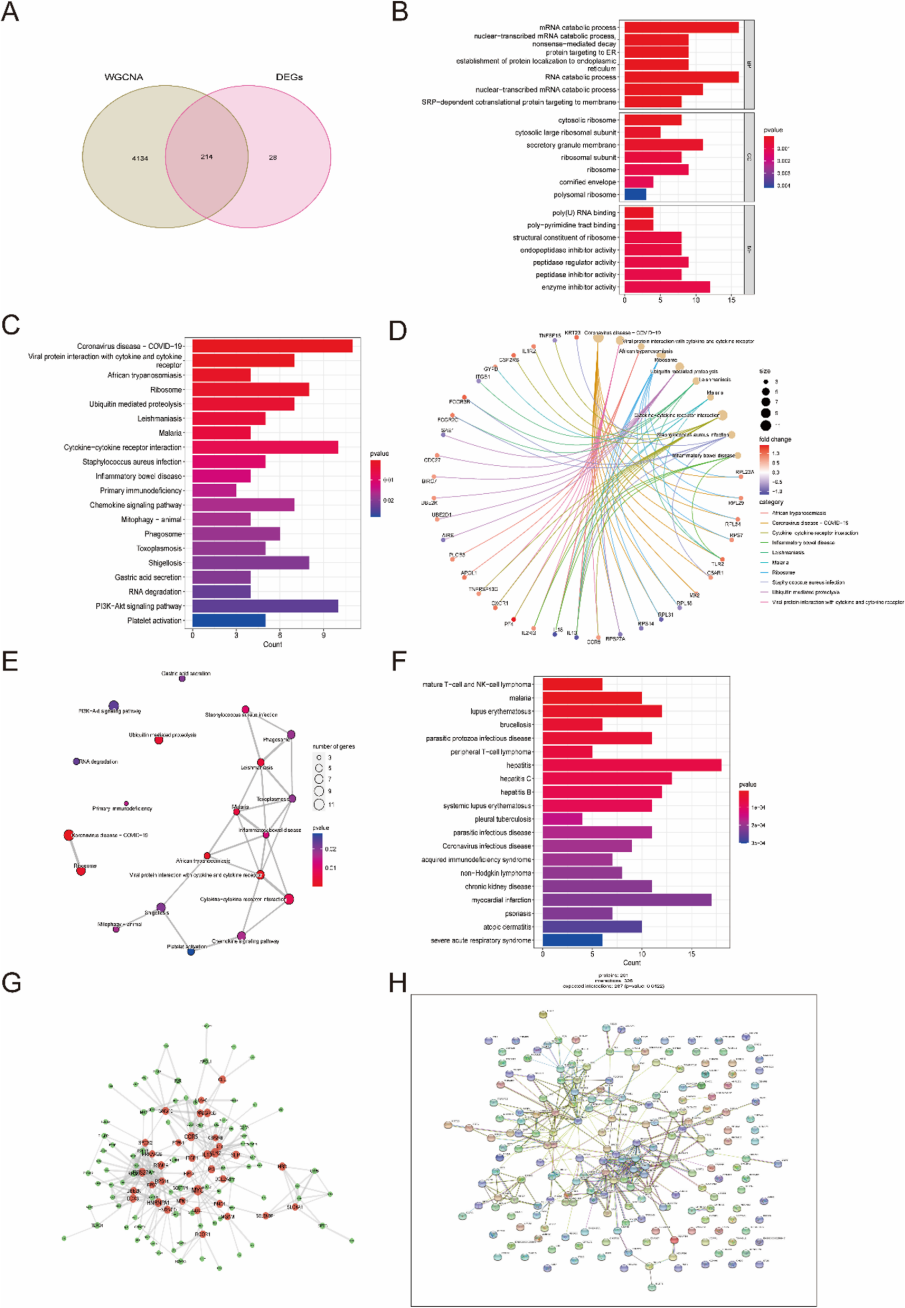
Functional enrichment analysis of overlapping genes. **A** Venn diagram showed the intersection of DEGs and module genes of WGCNA. **B**–**F** GO (**B**), KEGG (**C**–**E**), and DO (**F**) enrichment analysis of the overlapping genes. **G** Protein-Protein Interaction (PPI) network of overlapping genes. **H** The co-expression network showed the strength of correlation of hub genes from overlapping genes. Additionally, the appropriate copyright permission of these KEGG image was obtained and used in this study

A total of 214 candidate key genes were selected from the DEGs, yellow and turquoise modules (Supplementary Tables 6 and Fig. 4A). To explore the potential biological function and enrichment pathways, GO, KEGG, and DO analyses were performed on these candidate key genes. In the GO analysis, the candidate key genes were significantly enriched in the mRNA catabolic process, RNA catabolic process and nuclear-transcribed mRNA catabolic process for the biological process (BP) category. For the cellular component (CC) category, the genes were significantly enriched in cytosolic ribosome, secretory granule membrane and ribosome. The molecular function (MF) category was significantly enriched in poly-pyrimidine tract binding, endopeptidase inhibitor activity and enzyme inhibitor activity (Fig. 4B). In addition, the KEGG enrichment analysis showed that these overlapping genes were particularly related to ribosomes, ubiquitin-mediated proteolysis, leishmaniasis, malaria and cytokine-cytokine receptor interactions (Fig. 4C-E). The DO analysis showed that they were mainly enriched in hepatitis, systemic lupus erythematosus and malaria (Fig. 4F).

The results of these functional enrichment analyses indicated that SLE patients may share common immune processes with other diseases. To further reveal protein-protein interactions in SLE, PPI networks of candidate key genes were also analyzed (Fig. 4G, H). In general, these candidate key genes are closely related to each other and play pivotal roles in the pathogenesis of SLE.

### Further identification of optimal key genes through machine-learning algorithms

**Fig. 5:**
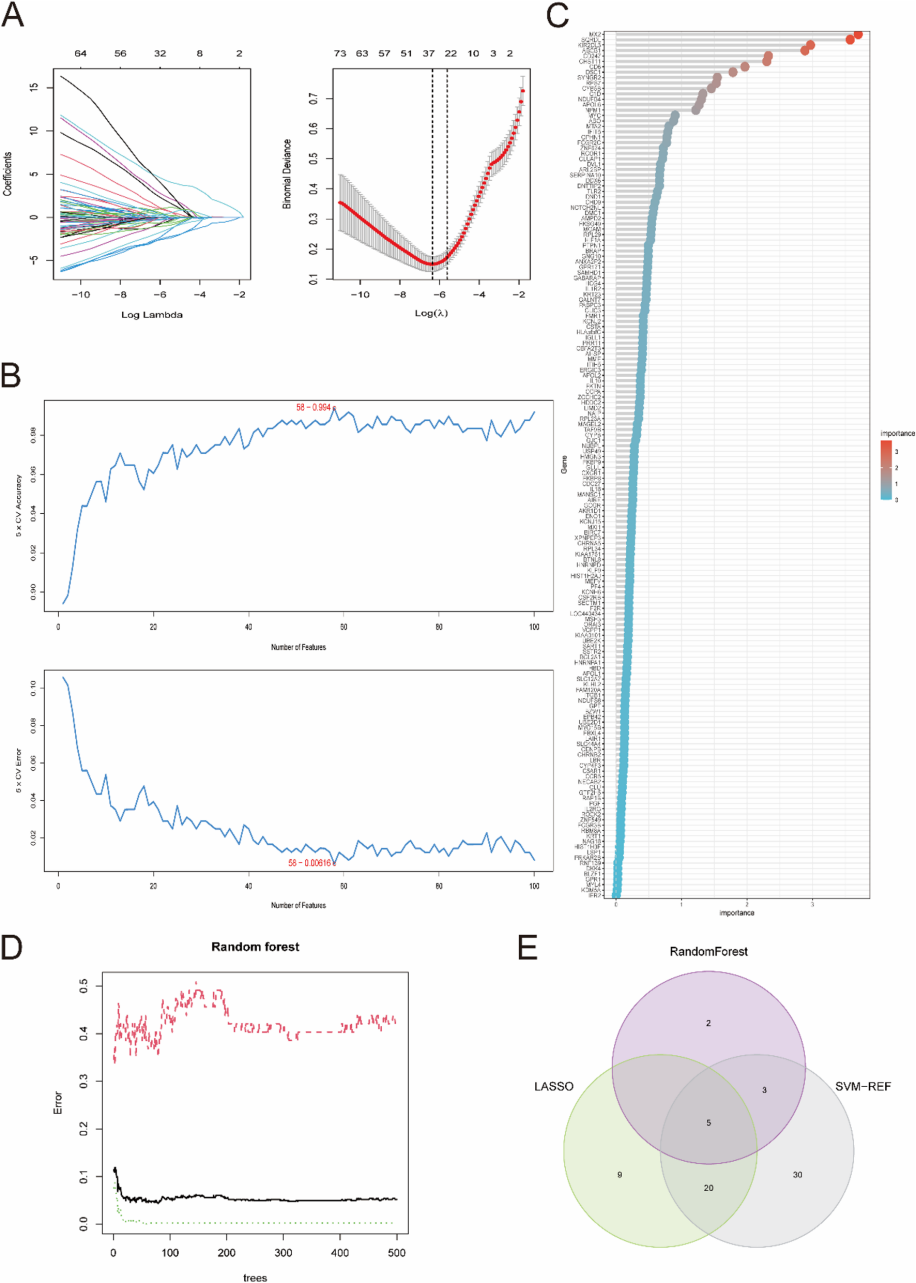
Three machine learning algorithms were applied to further explore the optimal key genes. **A** The LASSO algorithm determined the candidate optimal feature genes and the optimal lambda. Each coefficient curve in the left picture represents a single gene. The dashed vertical lines in the right picture represent the partial likelihood deviance. **B** The SVM-RFE algorithm was performed to further candidate optimal feature genes with the highest accuracy and lowest error obtained in the curves. **C** Top 10 key genes with importance > 2 were identified in the Random Forest algorithm. **D** Random forest for the relationships between the number of trees and error rate. **E** Venn diagram displayed the five optimal key genes overlapped by LASSO, Random Forest, and SVM-REF algorithms

To further explore the key genes, three machine-learning algorithms were performed. According to the 214 candidate key genes obtained above, 34 key genes were further selected as diagnostic markers of SLE by the LASSO algorithm (Fig. 5A and Supplementary Table 7). In addition, 58 key genes were screened after fivefold cross-validation of 214 genes using the SVM-REF algorithm (Fig. 5B and Supplementary Table 8). For the RF algorithm, the top 10 key genes with importance > 2 were identified, consisting of MX2, SQRDL, RFTN1, KIR2DL3, ABCB1, RPS14, CD247, CHST11, CD6 and DSC1 (Fig. 5C, D). Finally, all the key genes obtained by these machine-learning algorithms overlapped again, and the 5 optimal key genes were screened out, consisting of ABCB1, CD247, DSC1, KIR2DL3 and MX2, which were regarded as potential targets of SLE (Fig. 5E).

### Evaluation of the expression and diagnostic value of optimal key genes

**Fig. 6:**
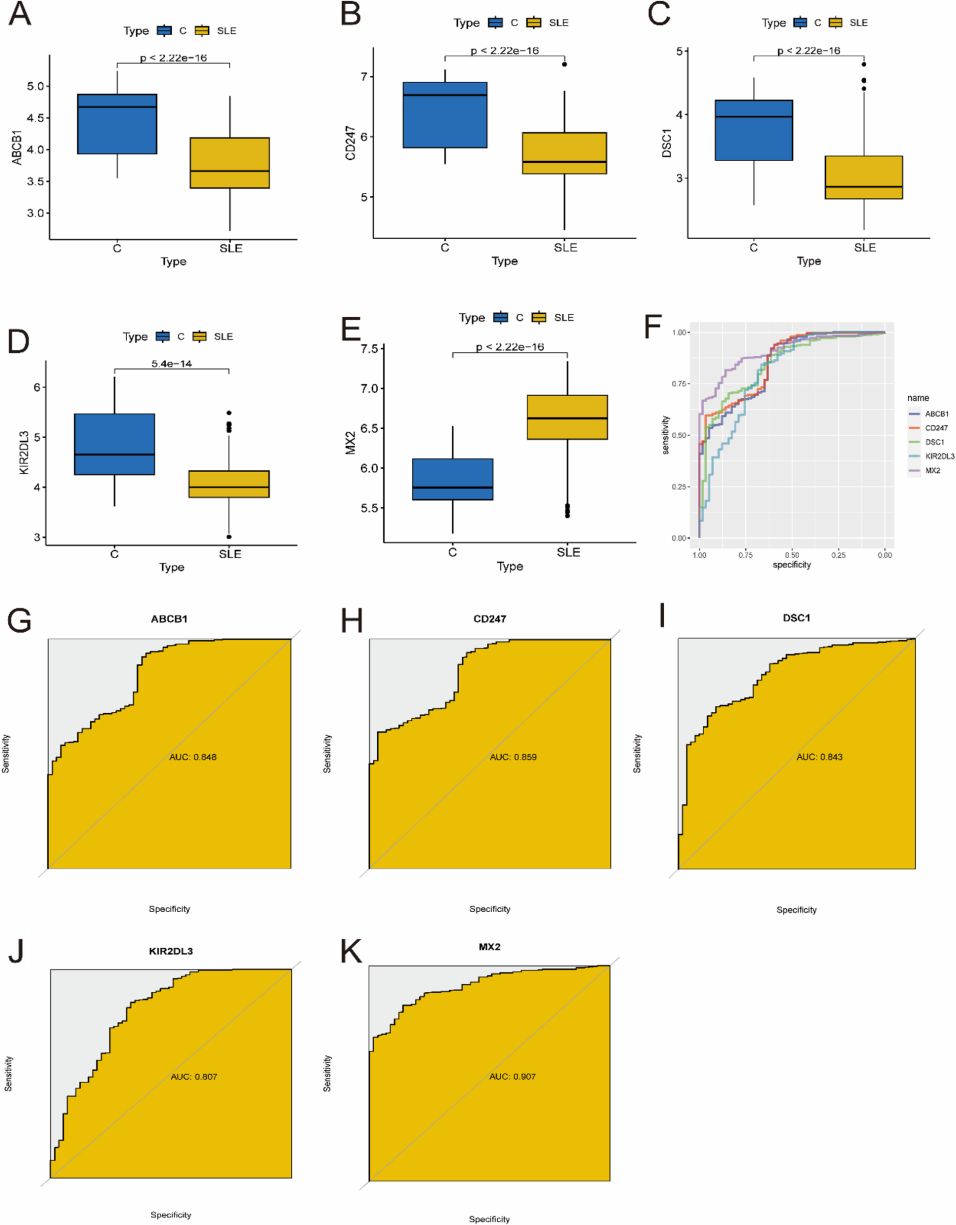
Verification of expression and diagnostic efficacy of optimal key genes in predicting SLE progression. **A**–**G** Box plots showing the expression of ABCB1 (**A**), CD247 (**B**), DSC1 (**C**), KIR2DL3 (**D**) and MX2 (**E**) in control and SLE samples. Statistic tests: Wilcoxon rank-sum test. **F**–**K** Roc curves (**F**) estimating the diagnostic performance of ABCB1 (**G**), CD247 (**H**), DSC1 (**I**), KIR2DL3 (**J**) and MX2 (**K**)

The expression of five optimal key genes was further analyzed in 425 SLE samples and 57 normal samples. In Fig. 6A-E, the expression of ABCB1, CD247, DSC1 and KIR2DL3 was significantly downregulated in SLE samples, while the expression of MX2 was significantly upregulated, indicating that they might have critical roles in the development of SLE (all *P* < 0.01). In addition, ROC curve analysis was performed to assess the diagnostic value of the optimal key genes (Fig. 6F). The AUC values of the ROC curves were 0.848 for ABCB1 (Fig. 6G), 0.859 for CD247 (Fig. 6H), 0.843 for DSC1 (Fig. 6I), 0.807 for KIR2DL3 (Fig. 6J), and 0.907 for MX2 (Fig. 6K), indicating that the five key genes have high diagnostic value in evaluating the progression of SLE.

**Fig. 7:**
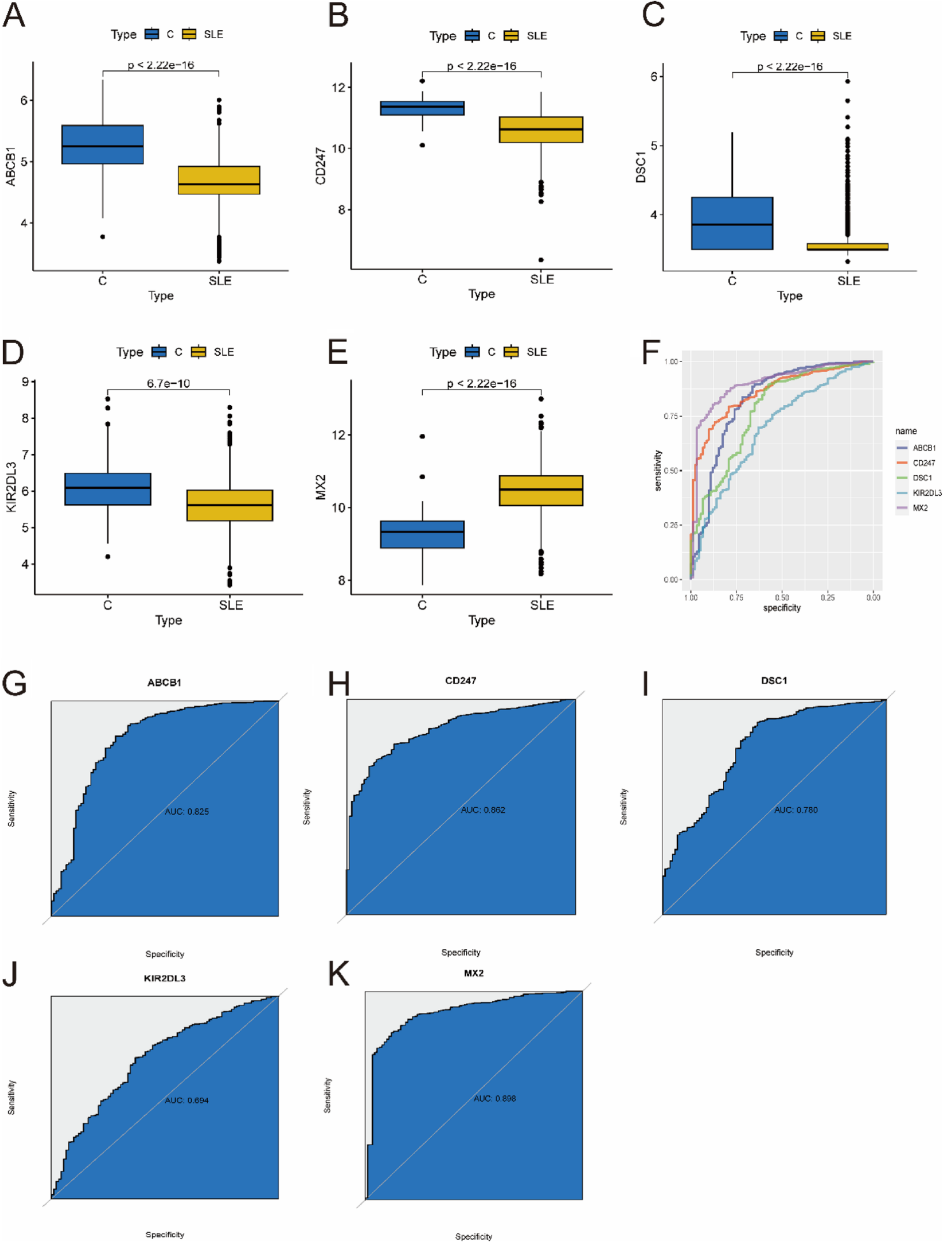
Verification of expression and diagnostic efficacy of optimal feature genes using external validation datasets. **A**–**G** Box plots showing the expression of ABCB1 (**A**), CD247 (**B**), DSC1 (**C**), KIR2DL3 (**D**) and MX2 (**E**) in control and SLE samples. Statistic tests: Wilcoxon rank-sum test. **F**–**K** Roc curves (**F**) estimating the diagnostic performance of ABCB1 (**G**), CD247 (**H**), DSC1 (**I**), KIR2DL3 (**J**) and MX2 (**K**)

To obtain more reliable results, the optimal key genes were further verified in an external validation dataset consisting of 1081 SLE samples and 92 control samples. Before analysis, the GSE65391 and GSE49454 datasets were normalized (Supplementary Fig. 1). Fortunately, the expression of 5 optimal key genes in SLE samples was consistent with the above results (Fig. 7A-E, all *P* < 0.05). The diagnostic value of these genes was assessed by ROC curve analysis (Fig. 7F). The AUC values of the external validation datasets were also high: ABCB1 (AUC: 0.825), CD247 (AUC: 0.862), DSC1 (AUC: 0.780), KIR2DL3 (AUC: 0.694), MX2 (AUC: 0.898) (Fig. 7G–K). Therefore, these results again prove that all the optimal key genes are related to SLE.

### Identification of the biological function of five key genes

**Fig. 8:**
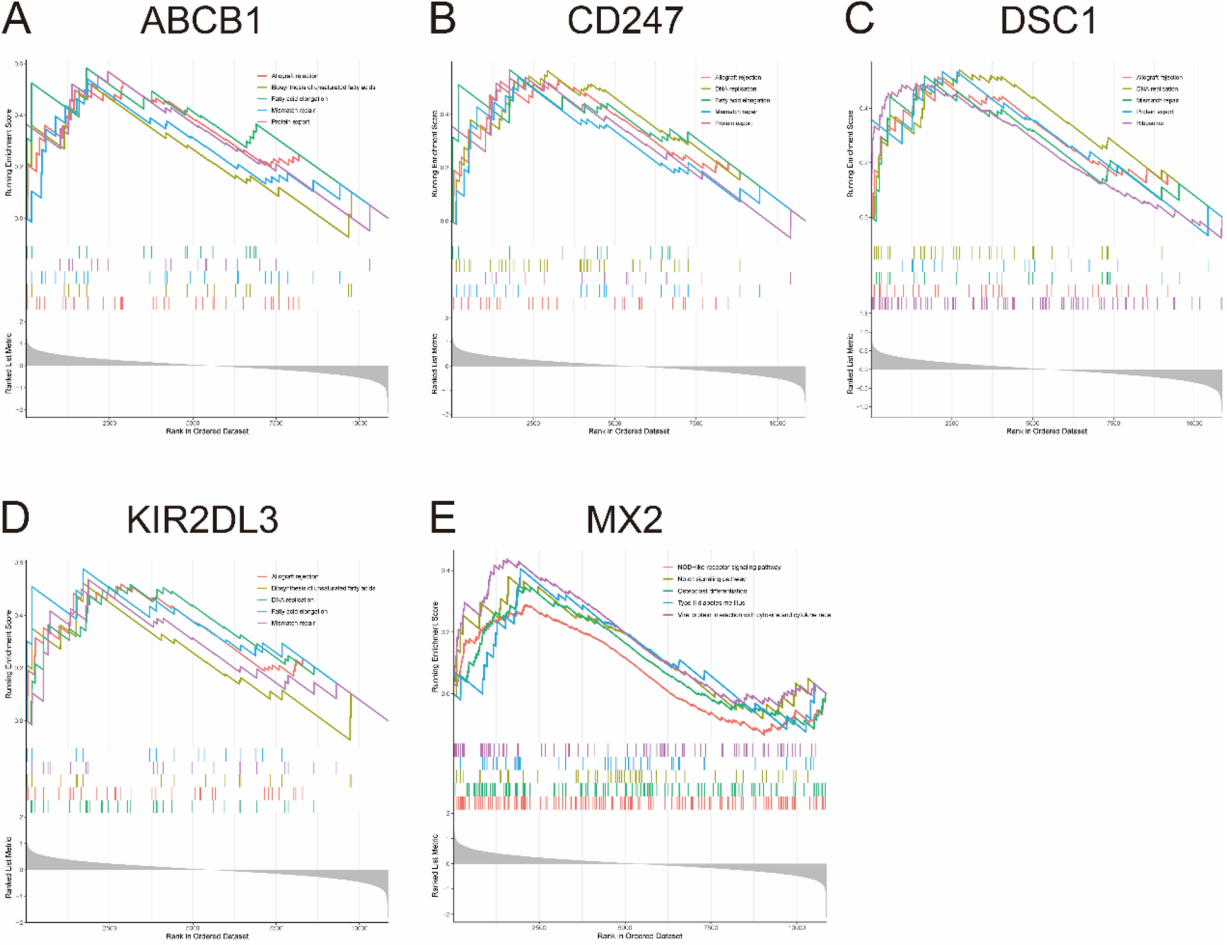
The GSEA analysis identifies signaling pathways in the five optimal key genes. **A**–**E** Top five signaling pathways that are significantly enriched in the high expression of ABCB1 (**A**), CD247 (**B**), DSC1 (**C**), KIR2DL3 (**D**) and MX2 (**E**)

To clarify the biological functions of the five optimal key genes, GSEA was employed. According to the median expression levels of these genes, SLE samples were segmented into two groups. In addition, immune-related pathways such as allograft rejection, mismatch repair, and protein export were significantly enriched in the high ABCB1 subgroup (Fig. 8A), but pathways such as osteoclast differentiation and type II diabetes mellitus were enriched in the low ABCB1 subgroup (Supplementary Fig. 2A). DNA replication, fatty acid elongation, mismatch repair, and protein export were significantly enriched in the high CD247 subgroup (Fig. 8B), but arachidonic acid metabolism and type II diabetes mellitus were significantly enriched in the low CD247 subgroup (Supplementary Fig. 2B). Allograft rejection, DNA replication, mismatch repair, protein export and ribosome were significantly enriched in the high DSC1 subgroup (Fig. 8C), but glycerophospholipid metabolism, notch signaling pathway, and viral protein interaction with cytokine and cytokine receptor were significantly enriched in the low DSC1 subgroup (Supplementary Fig. 2C). Allograft rejection, fatty acid elongation, and mismatch repair were significantly enriched in the high KIR2DL3 subgroup (Fig. 8D), but measles, type II diabetes mellitus, and viral protein interaction with cytokine and cytokine receptor were significantly enriched in the low KIR2DL3 subgroup (Supplementary Fig. 2D). The NOD-like receptor signaling pathway, notch signaling pathway and osteoclast differentiation were significantly enriched in the high MX2 subgroup (Fig. 8E), but fatty acid elongation, mismatch repair and protein export were enriched in the low MX2 subgroup (Supplementary Fig. 2E).

### Immune cell infiltration analysis

**Fig. 9:**
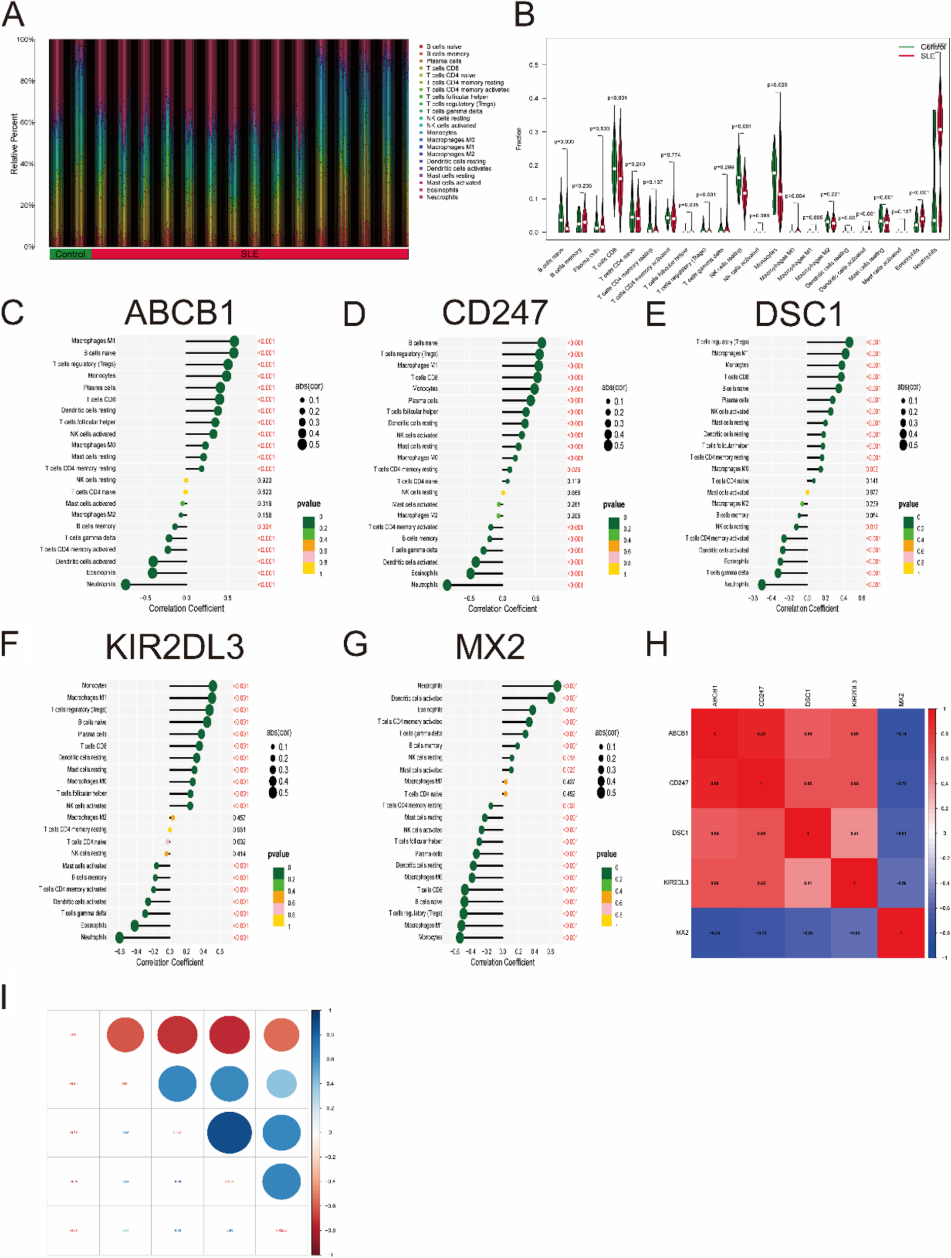
Analysis of immune cell infiltration. **A** The relative percent of 22 immune cells types between control samples and SLE samples. **B** Boxplot shows the differences of infiltrated immune cells between control samples and SLE samples. Statistic tests: Wilcoxon rank-sum test. (*P* < 0.05*; *P* < 0.01**; *P* < 0.001***; ns, no significance). **C**–**G** Correlation between immune cells and optimal key genes ABCB1 (**C**), CD247 (**D**), DSC1 (**E**), KIR2DL3 (**F**) and MX2 (**G**). **H**, **I** Correlation analysis of five optimal key genes in SLE samples

The CIBERSORT algorithm was used to evaluate the differences in immune cell infiltration and hallmark gene sets between the SLE and control groups. As shown in Fig. 9A, B, the proportions of naive B cells, regulatory T cells (Treg cells), monocytes, M0/M1 macrophages, eosinophils and neutrophils in SLE samples were significantly increased compared with those in control samples, while the percentages of CD8 T cells, NK cells, dendritic cells and resting mast cells were significantly decreased.

Furthermore, correlation analysis revealed that four optimal key genes (ABCB1, CD247, DSC1, and KIR2DL3) were significantly positively related to the infiltration of monocytes, M0/M1 macrophages, Treg cells, CD8 T cells, resting dendritic cells and resting mast cells but negatively related to the infiltration of activated memory CD4 T cells, activated dendritic cells, eosinophils and neutrophils (Fig. 9C-F). However, the MX2 gene is roughly the opposite of the above four genes (Fig. 9G). For instance, the ABCB1 gene was positively related to M1 macrophages (*R* = 0.58, *P* < 2.2e-16) but negatively related to neutrophils (R = − 0.74, *P* < 2.2e-10) (Supplementary Fig. 3). In Fig. 9H, I, gene correlations were also performed. These five optimal key genes displayed significant correlations. For example, the correlation coefficient between ABCB1 and CD247 was 0.88, but the correlation coefficient between ABCB1 and MX2 was − 0.74, indicating that four optimal key genes (ABCB1, CD247, DSC1 and KIR2DL3) had a significant functional similarity, while the function of MX2 gene was opposite to the remaining four genes.

**Fig. 10:**
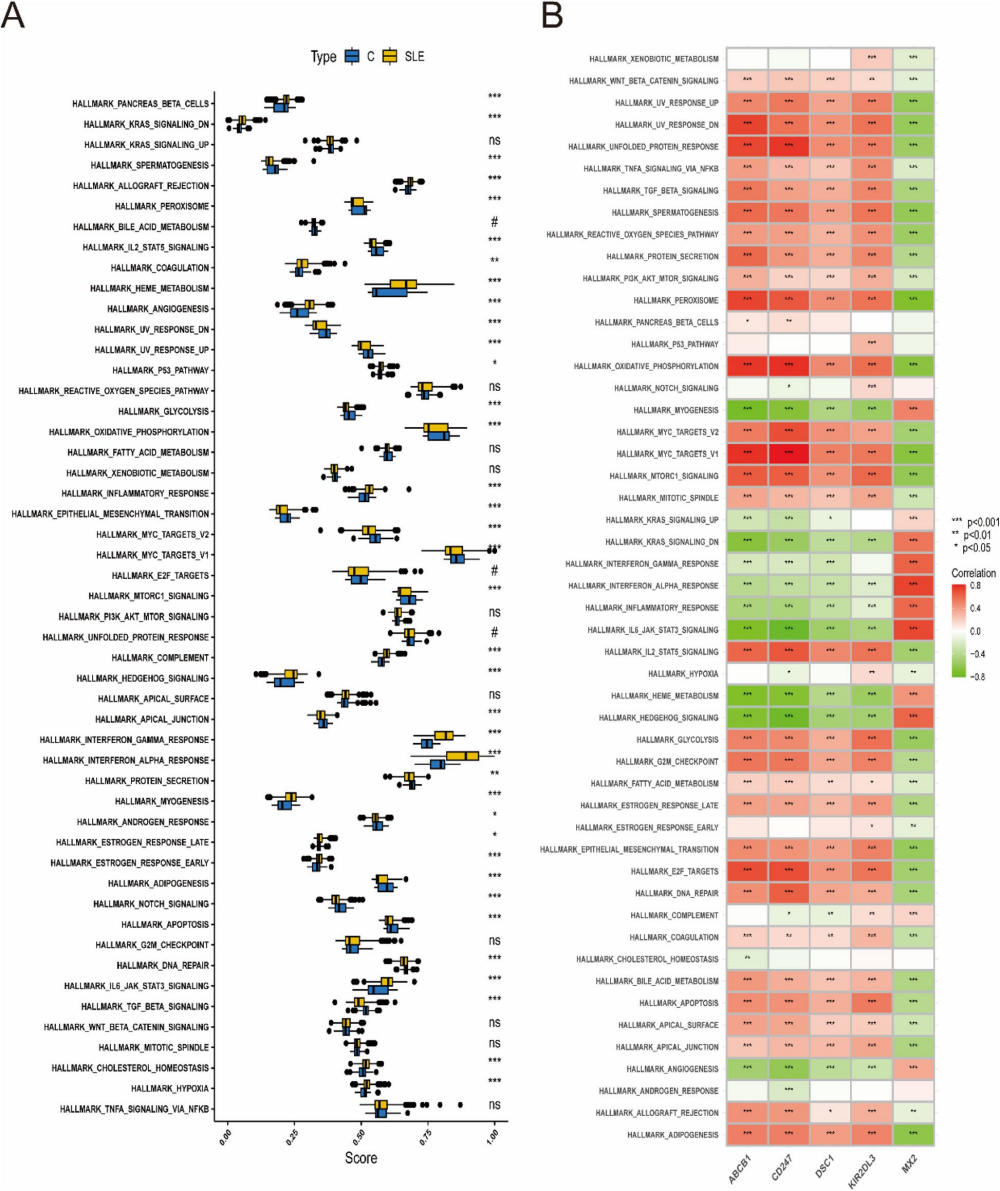
Analysis of hallmark gene sets. **A** The specific distribution of the 50 hallmark gene sets in SLE and control samples. **B** Correlation analysis of the 50 hallmark gene sets with five optimal key genes. Statistic tests: Wilcoxon rank-sum test (*P* < 0.05*; *P* < 0.01**; *P* < 0.001***; ns, no significance)

To explore whether there were differences in the hallmark gene sets between the SLE and control groups, the ssGSEA algorithm was used to identify the significance of differences in 50 hallmark gene sets between the two groups according to the enrichment score. The distribution between the SLE and control groups is shown in Fig. 10A.

The gene sets showed significant differences, such as allograft rejection, coagulation, heme metabolism, angiogenesis, P53 pathway, inflammatory response and hypoxia. Hence, compared with the control group, these hallmark gene sets might be overactivated in the SLE group. In addition, four optimal key genes except for MX2 were generally consistent. For example, the four optimal key genes (ABCB1, CD247, DSC1, KIR2DL3) were positively related to the oxidative phosphorylation hallmark gene set, while MX2 was negatively related to it (Fig. 10B). Therefore, the role of the above genes should be further explored in SLE. 

### Validation of the five optimal key genes

**Fig. 11:**
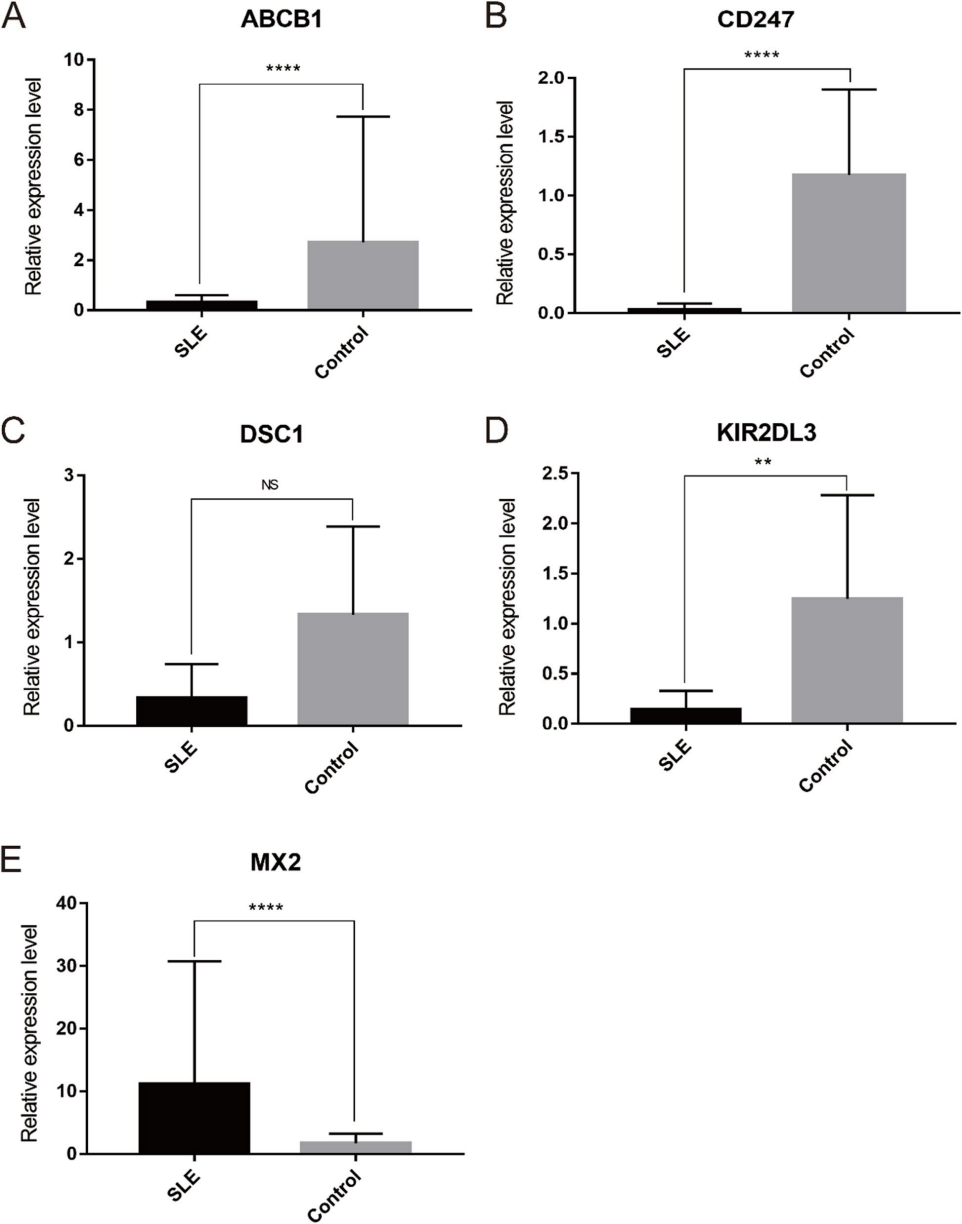
The relative expressions of optimal key genes were validated by RT-qPCR. **A**–**E** The expressions of ABCB1 (A), CD247 (B), DSC1 (**C**), KIR2DL3 (**D**) and MX2 (**E**) between SLE patients and healthy control. Statistic tests: Student’s t-test (*P* < 0.01**; *P* < 0.001***; *P* < 0.0001****; ns, no significance)

The relative expression of five optimal key genes was verified with RT-qPCR in SLE patients and healthy controls. Compared with the control group, the expression of ABCB1 (Fig. 11A), CD247 (Fig. 11B), and KIR2DL3 (Fig. 11D) was significantly downregulated, while MX2 (Fig. 11E) was significantly upregulated in SLE patients (all *P* < 0.05), which was consistent with the results of this bioinformatic analysis. However, the expression of DSC1 (Fig. 11C) was also downregulated in SLE samples, although there was no statistical significance (*P* >0.05).

## Discussion

SLE is an autoimmune-mediated diffuse connective tissue disease characterized by immune inflammation that involves multiple systems and organs of the whole body. The main clinical characteristics are immune disorder and systemic inflammation, leading to progressive and irreversible multiorgan damage [34]. The clinical manifestations are extensive, including kidney, dermatological, neuropsychiatric, and cardiovascular symptoms. For many years, the pathogenesis of SLE has been the focus of international research. The current medical consensus is that the pathogenesis of SLE is the result of the interaction of genetic, environmental and random factors. Autoantibodies, including anti-dsDNA antibodies and anti-SM antibodies, are of great significance for the diagnosis of SLE, but the sensitivity is low. The existing SLE immune serological indices cannot reach high sensitivity and specificity at the same time, resulting in difficulties in the diagnosis of SLE. Therefore, it is urgent to explore new biological markers to improve the diagnostic rate of SLE.

In recent years, bioinformatic technology has been increasingly developed in the fields of medical research, such as the search for pathogenic genes and the screening of effective drug targets. In this study, three datasets were downloaded from GEO databases for the subsequent bioinformatic analysis, and 214 candidate genes were obtained after overlapping the genes analyzed by the DEG and WGCNA. Then, functional enrichment analysis was performed to explore the potential biological function and enrichment signaling pathways. As shown in Fig. 4, these candidate genes are related to the PI3K-Akt signaling pathway. Previous studies have shown that the occurrence of SLE may be related to the up-regulation of the PI3K-Akt-mTOR pathway [35]. The DO analysis revealed the candidate genes were mainly enriched in systemic lupus erythematosus. Moreover, the PPI network of candidate key genes displayed these candidate key genes are closely related to each other, and previous studies suggested that these genes are involved in the occurrence and development of SLE [36, 37]. Therefore, further analysis of these candidate genes is significant. To further explore the feature genes, three machine-learning algorithms were performed, including the LASSO, RF and SVM-REF. Finally, the five optimal feature genes were screened out, consisting of ABCB1, CD247, DSC1, KIR2DL3 and MX2. Subsequently, the expression and diagnostic value of these genes were evaluated. The expression of ABCB1, CD247, DSC1 and KIR2DL3 was significantly downregulated in SLE samples, while the expression of MX2 was significantly upregulated. The ROC curve analysis confirmed that these genes were highly expressed in SLE samples in the above datasets. Furthermore, GO, KEGG, DO and GSEA analyses were used to study the biological functions of these genes. ssGSEA was carried out to assess differences in immune cell infiltration and hallmark gene sets between SLE and control samples.

To obtain more reliable results, the expression and diagnostic value of the five optimal feature genes were further verified in an external validation dataset which was also obtained from the GEO database, and the results confirmed by this method were consistent with the previous results. In addition, RT-qPCR was performed to analyze the relative expression of the five optimal key genes in SLE and healthy control samples, and the results showed that except for DSC1, the expressions of other genes (ABCB1, CD247, KIR2DL3 and MX2) were consistent with the results of biological information analysis. This deviation may be related to the number of samples, we will expand the sample size in follow-up studies to further confirm these findings based on the gene and protein levels.

At present, several studies have discussed the relationship between these genes and the pathogenesis of SLE [38–43]. ABCB1 is a multidrug resistant protein mainly expressed in the brain, liver and skin, as well as in other organs [44]. Recently, ABCB1 has been reported to be expressed in a variety of immune cells, such as monocytes, antigen-presenting dendritic cells, T cells, and B cells, and is involved in the expulsion of inflammatory molecules [45, 46]. The expression level of ABCB1 is reported to be significantly lower in SLE patients than in controls [40]. CD247, also known as the T-cell surface glycoprotein CD3 zeta chain, is one part of the T-cell antigen receptor (TCR) complex and plays a critical role in receptor expression and signaling. Studies have shown that low CD247 expression due to chronic inflammation is related to the decreased T-cell activity [47]. Accordingly, the expression of CD247 is significantly lower in SLE patients than in healthy controls [41]. Killer cell immunoglobulin-like receptor (KIR) is expressed on the surface of human natural killer cells (NK cells) and T cells and is associated with viral infection and tumor and autoimmune diseases. KIR2DL3 plays an important role in some immune-related diseases [48, 49]. Meanwhile, the KIR2DL3 gene was also significantly lower in SLE patients than in healthy subjects [42]. MX2 is an interferon inducible gene that is mostly known for its antiviral activity [50]. MX2 exerts immune effects by mediating the effects of multiple immune cells, including neutrophils, macrophages and T cells [51]. In addition, significantly high expression of MX2 was also found in SLE samples [43]. However, studies on DSC1 and SLE have not been found. To make them more credible, RT-qPCR results showed that the relative expression of ABCB1, CD247, and KIR2DL3 was significantly downregulated, while MX2 was significantly upregulated in SLE patients. Notably, DSC1 was also lower in SLE samples, but without statistical significance. In general, the validation results were in line with the bioinformatic analysis and previous studies.

In the analysis of immune cell infiltration, different types of immune cells, including naive B cells, regulatory T cells (Treg cells), monocytes, plasma cells and NK cells, were significantly regulated in SLE samples. Subsequently, the correlations between immune cells and the five optimal key genes were performed. Four optimal key genes (ABCB1, CD247, DSC1, and KIR2DL3) were found to have roughly the opposite correlation to MX2 with infiltration of several immune cells. Meanwhile, in GSEA analysis, the four optimal key genes (ABCB1, CD247, DSC1 and KIR2DL3) have the opposite effects on many signaling pathways as MX2. For instance, the four optimal key genes (ABCB1, CD247, DSC1, KIR2DL3) were positively related to the PI3K-Akt-mTOR signaling pathway, while MX2 was negatively related to it. This characteristic is consistent with the relationship between the five feature genes and immune cells, which means that these genes might affect the signaling pathways by acting on these immune cells.

This study relies on bioinformatics analysis, and there are some inconsistencies in the results. More in vivo and in vitro experiments should be provided to verify the results.

## Conclusion

In this study, a total of five optimal key genes were screened, providing new targets for the pathogenesis of SLE. Additionally, more machine learning algorithms were applied to improve the accuracy of genetic screening. Moreover, this study not only used external dataset verification but also carried out RT-qPCR experiment verification, making the screening results more reliable. In conclusion, the purpose of this study is to provide a new direction for the clinical diagnosis and precise treatment of SLE by machine-learning analysis.

### Supplementary Information

**Additional file 1: Table S1. **The information of datasets.

**Additional file 2: Table S2. **Sequences of Primers for the real-time qPCR.

**Additional file 3: Table S3. **242 DEGs between control normal and SLE samples.

**Additional file 4: Table S4. **GSEA results.

**Additional file 5: Table S5. **4348 candidate genes from the yellow and turquoise modules.

**Additional file 6: Table S6. **214 overlapping genes from WGCNA and DEGs.

**Additional file 7: Table S7. **34 candidate feature genes from LASSO algorithm.

**Additional file 8: Table S8.** 58 feature genes from SVM-REF algorithm.

**Additional file 9: Supplementary Figure 1. **Box plot of external validation dataset after normalization. **Supplementary Figure 2. **The GSEA analysis identifies signaling pathways in the five optimal key genes. **Supplementary Figure 3. **The relationship between optimal key genes and immune cells

## Data Availability

The original contributions presented in the study are included in the article/ supplementary material. Further inquiries can be directed to the corresponding authors.

## Abbreviations

BP: Biological process
CC: Cellular component
DEGs: Differentially expressed genes
DO: Disease Ontology
GEO: Gene Expression Omnibus
GO: Gene Ontology
GS: Gene importance
GSEA: Gene set enrichment analysis
KEGG: Kyoto Encyclopedia of Genes and Genomes
KIR: Killer cell immunoglobulin-like receptor
LASSO: Least absolute shrinkage and selection operation (LASSO)
MF: Molecular function
MM: Module membership
NK cells: Natural killer cells
PPI: Protein-protein interaction
RF: Random forest
ROC: Receiver operating characteristic curves
RT-qPCR: Real-time quantitative polymerase chain reaction
SLE: Systemic lupus erythematosus
SVM-REF: Support vector machine-recursive feature elimination
TCR: T cell antigen receptor
TOM: Topological overlap matrix
WGCNA: Weighted gene coexpression network
