# Supplementary material for: Exploration of biomarkers for systemic lupus erythematosus by machine-learning analysis
Source: BMC Immunol. 2023 Nov 10;24:44. doi: 10.1186/s12865-023-00581-0 (PMC10638835; doi:10.1186/s12865-023-00581-0)
Supplement: Supplementary file 9 — Additional file 9: Supplementary Figure 1. Box plot of external validation dataset after normalization. Supplementary Figure 2. The GSEA analysis identifies signaling pathways in the five optimal key genes. Supplementary Figure 3. The relationship between optimal key genes and immune cells [file 12865_2023_581_MOESM9_ESM.docx]

**Supplementary Materials**

**
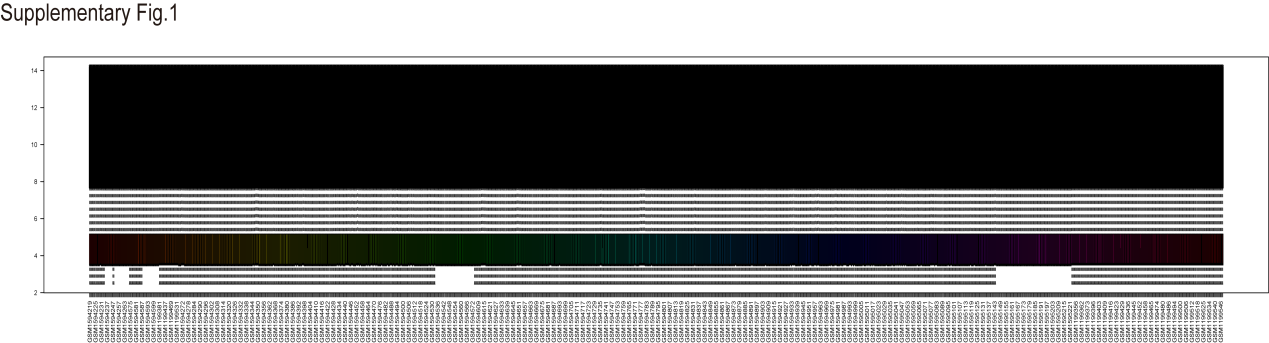
**

**Supplementary Figure 1. Box plot of external validation dataset after normalization.**


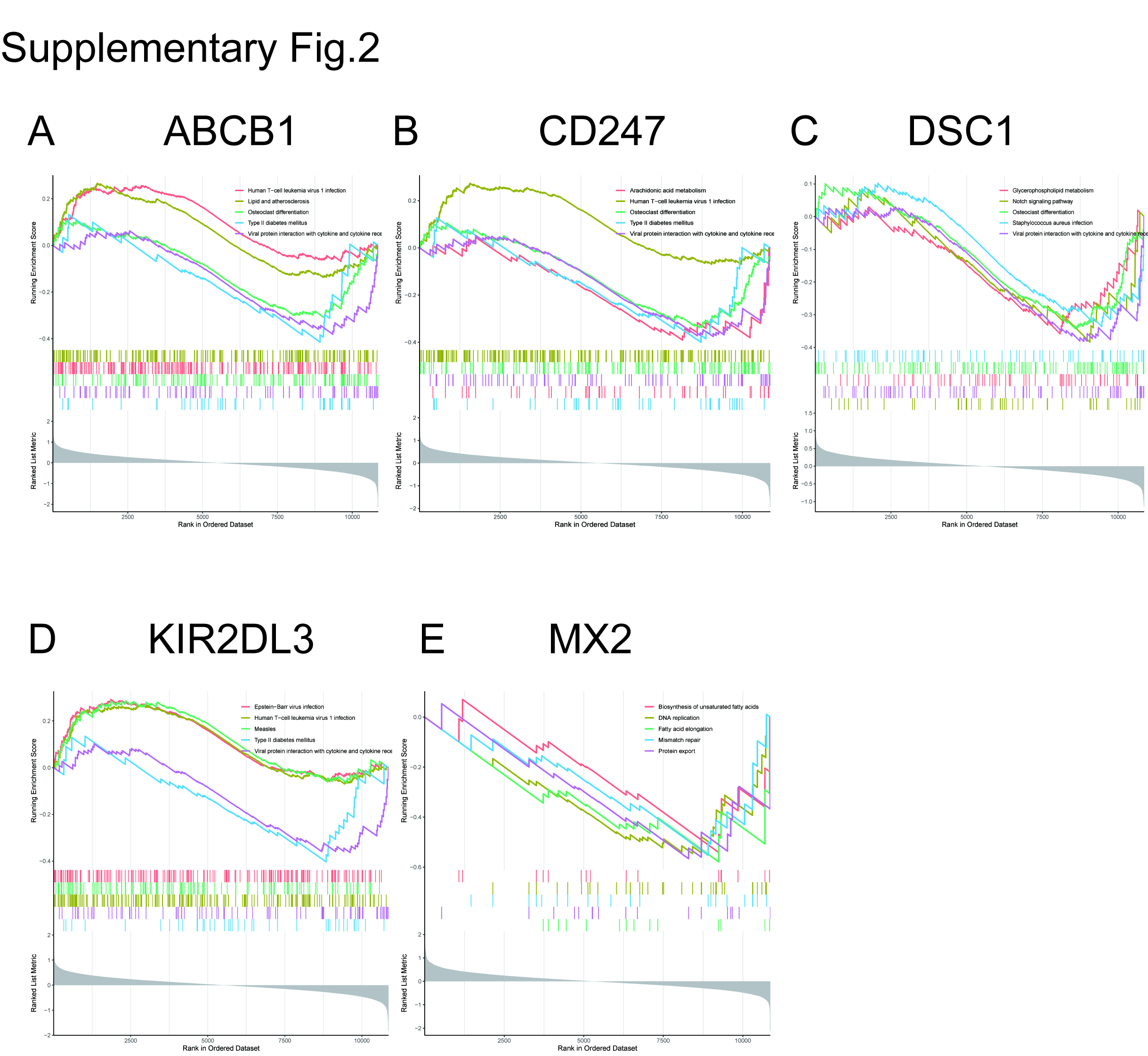


**Supplementary Figure 2. The GSEA analysis identifies signaling pathways in the five optimal key genes.** (A–E) Top five signaling pathways that are significantly enriched in the low expression of ABCB1 (A), CD247 (B), DSC1 (C), KIR2DL3 (D) and MX2 (E).

**Supplementary Figure 3. The relationship between optimal key genes and immune cells.** Positive relationship between ABCB1 (A), CD247 (C), DSC1 (F), KIR2DL3 (G), MX2 (J) and immune cells. Negative relationship between ABCB1 (B), CD247 (D), DSC1 (E), KIR2DL3 (H), MX2 (I) and immune cells.
